# The Diagnosis and Treatment of Some of the Common Diseases of the Rectum and Anus

**Published:** 1905-09

**Authors:** 


**The Diagnosis and Treatment of some of the Common Diseases of the Rectum and Anus.** By CECIL H. LEAF, M.A., M.B. Pp. viii., 118. London: E. H. Blakeley. 1905. **Surgical Case Book Charts for Rectal Diseases.** By CECIL H. LEAF, M.A., M.B. London: Archibald Constable & Co. Ltd.

This treatise on diseases of the rectum and anus is a useful and practical elementary manual from which many helpful facts may be gleaned. It deals with surgical anatomy and the more common diseases and their treatment. We note that the author prefers to examine the patient in the semi-prone position on the *right* side, and that in the treatment of fissure of the anus he does not divide the base of the ulcer but the sphincter laterally, and gives reasons for avoiding section in the middle line anteriorly or posteriorly. With regard to the treatment of cancer of the rectum by colostomy, the author thinks that the operation should always be performed when the passage of fæces causes distress and before signs of obstruction become manifest. In excision of the rectum for cancer, he holds that colostomy should always be performed, and that the artificial anus thus made should be a permanent one. We warmly recommend the perusal of this book. The charts designed for rectal diseases may be of service to those who are not draughtsmen.

